# DNA methylation profiling reveals common signatures of tumorigenesis and defines epigenetic prognostic subtypes of canine Diffuse Large B-cell Lymphoma

**DOI:** 10.1038/s41598-017-11724-w

**Published:** 2017-09-14

**Authors:** Serena Ferraresso, Arianna Aricò, Tiziana Sanavia, Silvia Da Ros, Massimo Milan, Luciano Cascione, Stefano Comazzi, Valeria Martini, Mery Giantin, Barbara Di Camillo, Sandro Mazzariol, Diana Giannuzzi, Laura Marconato, Luca Aresu

**Affiliations:** 1 Department of Comparative Biomedicine and Food Science, University of Padova, Legnaro (PD), Italy; 2 Department of Biomedical Informatics, Harvard Medical School, Boston, MA USA; 3 Lymphoma & Genomics Research Program, Institute of Oncology Research, Bellinzona, Switzerland; 4 Oncology Institute of Southern Switzerland, Bellinzona, Switzerland; 5 Department of Veterinary Medicine, Università degli Studi di Milano, Milano (MI), Italy; 6 Department of Information Engineering, University of Padova, Padova (PD), Italy; 7 Centro Oncologico Veterinario, Bologna, Italy

## Abstract

Epigenetic deregulation is a hallmark of cancer characterized by frequent acquisition of new DNA methylation in CpG islands. To gain insight into the methylation changes of canine DLBCL, we investigated the DNA methylome in primary DLBCLs in comparison with control lymph nodes by genome-wide CpG microarray. We identified 1,194 target loci showing different methylation levels in tumors compared with controls. The hypermethylated CpG loci included promoter, 5′-UTRs, upstream and exonic regions. Interestingly, targets of polycomb repressive complex in stem cells were mostly affected suggesting that DLBCL shares a stem cell-like epigenetic pattern. Functional analysis highlighted biological processes strongly related to embryonic development, tissue morphogenesis and cellular differentiation, including HOX, BMP and WNT. In addition, the analysis of epigenetic patterns and genome-wide methylation variability identified cDLBCL subgroups. Some of these epigenetic subtypes showed a concordance with the clinical outcome supporting the hypothesis that the accumulation of aberrant epigenetic changes results in a more aggressive behavior of the tumor. Collectively, our results suggest an important role of DNA methylation in DLBCL where aberrancies in transcription factors were frequently observed, suggesting an involvement during tumorigenesis. These findings warrant further investigation to improve cDLBCL prognostic classification and provide new insights on tumor aggressiveness.

## Introduction

Canine diffuse large B-cell lymphoma (cDLBCL) is the most frequent malignancy of B-lymphocytes in dog and comprises approximately 60–70% of all cases. Dose-intense chemotherapy, bone marrow transplantation and immunotherapy have emerged as the treatments of choice, but current therapeutic strategies are associated with short survival and high relapse rates^1^.

Recently, many efforts have been devoted to characterize, in a comprehensive way, the biological bases of cDLBCL pathogenesis. Two different comparative gene expression studies^2, 3^ demonstrated that cDLBCL shares similar features with its human counterpart, in particular highlighting the interplay among specific molecular pathways (i.e. NF-κB, PI3K/AKT, Notch and JAK/STAT), which may have potential therapeutic implications^2, 4^. In addition, recurrent copy number variations were identified by array comparative genomic hybridization (aCGH) including gains in chr13, syntenic to the region in human chromosome 8 containing *MYC* oncogene, and chr31^5–7^.

However, none of these gene signatures have been translated into clinic, suggesting the need for more robust molecular and prognostic studies. In medical research, evidence showed that genetic and genomics alone are not sufficient to explain the biological variability of cancer. The ability of epigenetic mechanisms to drive cells with the same genome towards different phenotypic identities represents one of the best promises in cancer research^8^. Normal epigenetic processes are usually disrupted during the initiation and progression of cancer and aberrant DNA methylation within CpG islands (CpGIs) is the first critical mark of epigenetic modifications affecting regulatory genomic regions in tumor cells. Hypermethylation of gene promoter regions commonly leads to transcriptional silencing while DNA methylation changes in CpG-poor regions (i.e. genic/intergenic) can play a critical role in the regulation of gene activity and genomic stability^9^.

In human, large-scale genomic studies have shown that perturbations of epigenetic patterning are frequent events in B-cell lymphoma^10^. The characterization of lymphoma methylation signatures and the understanding of how their changes contribute to cancer phenotype have paved the way to new therapeutic approaches in this tumor. Thus, nothing is known about the DNA methylome of canine B-cell lymphoma yet. The epigenetic changes driving B-cell lymphoma have been scarcely investigated in dog and current studies on aberrant methylation patterns focused only on single genes^11–15^. Here, genome-wide characterization of cDLBCL epigenome was performed through a design of a CpG microarray platform targeting more than 40,000 CpG regions and coding sequences (CDS) distributed across the entire dog genome. Then, we hypothesized that DNA methylation patterning in cDLBCLs can provide clues about gene deregulation by identifying aberrantly methylated genes and explaining the different clinical behavior of cDLBCL.

## Results

### Clinical characteristics of DLBCL dogs

Investigated dogs were composed by 32 (82.1%) purebred and 7 (17.9%) crossbred dogs. Among purebred dogs, German shepherds (n = 5, 15.6%), Dobermann dogs (n = 3, 9.3%) and Golden retrievers (n = 3, 9.3%) were the most common. There were 22 (56.4%) females and 17 (43.6%) males. Median age was 7 years (mean, 7.5; range, 3–13 years), and median weight was 25.7 kg (mean, 28.4 kg; range, 6.1–69 kg). Regarding clinical stage, 3 (7.7%) dogs had stage III disease, 16 (41%) dogs had stage IV disease, and 20 (51.3%) dogs had stage V disease. Among dogs with stage V disease, 15 (75%) had bone marrow involvement, 3 (15%) had lung involvement, 1 (5%) had cutaneous involvement, and 1 (5%) had peripheral blood involvement. At the time of diagnosis, 27 (69.2%) dogs were asymptomatic (substage a), whereas 12 (30.8%) dogs showed clinical signs (substage b). Overall, 15 (38.5%) dogs received prednisone at the dose of 0.5–1 mg/kg before being referred. All dogs were treated with the same dose-intense chemotherapeutic (CH) protocol, consisting of L-asparaginase (week 1), Vincristine (weeks 2, 3, 4 and 13), cyclophosphamide (weeks 2 and 13), doxorubicin (weeks 7 and 16), lomustine (weeks 10 and 19), and prednisone (weeks 1 through 20). 23 dogs also received an intradermal injection of an autologous vaccine (VAX) on weeks 4, 5, 6, 7, 12, 16, 20 and 24. 20 (51.3%) dogs relapsed while being treated, whereas in 14 (35.9%) dogs lymphoma recurred after the end of treatment. 5 (12.8%) dogs never relapsed and were still in first complete remission at data analysis closure. Median TTP for all dogs was 162 days (range 1–1174). Median LSS was 281 days (range 12–1175 days), with 1 dog being alive at data analysis closure. Complete clinical features of dogs are reported in Supplementary File 1 (Table S1).

### Identification of loci differentially methylated between cDLBCL and control lymph nodes

**Figure 1:**
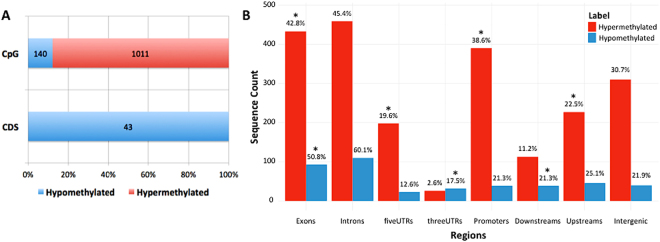
Genomic distribution of differentially methylated sequences in cDLBCLs versus control lymph nodes. (**A**) Percentages of hyper- and hypo-methylated probes across CpG regions and CDS. Corresponding sequence counts are reported in each barplot. (**B**) Distribution of hyper- and hypo-methylated features across different genomic locations. Percentages with respect to the corresponding total number of hyper- and hypo-methylated sequences are reported. Sequence counts allow repetitions, since a sequence can overlap more than one genomic location. Asterisks (*) indicate enriched genomic locations, according to Fisher’s Exact test (Supplementary File 1).

Both raw and normalized methylation data are available at the Gene Expression Omnibus (GEO) repository under accession number GSE94913 (AN: data are kept private until manuscript acceptance, however, for those interested, a reviewer access link has been provided to the editor). Analysis of differential methylated regions (DMRs) focused on 29,513 sequences (29,298 CpG regions and 215 CDS) after filtering out sequences with low methylation. Mann-Whitney Wilcoxon test identified 1,011 hypermethylated CpG regions and 183 hypomethylated sequences (140 CpG and 43 CDS regions) in cDLBCLs (n 37) versus control (n 7) lymph nodes (Fig. 1A and Table S2). These sequences were annotated to genomic regions associated to 823 genes. Interestingly, hyper-methylated sequences were enriched for promoter, 5′-UTRs, upstream and exonic regions (Bonferroni-adjusted p-values < 0.006), whereas hypo-methylated sequences were enriched for exonic, 3′-UTRs and downstream regions (Bonferroni-adjusted p-values < 0.0005, Fig. 1B).

### Differentially methylated genes are involved in key pathways of development and morphogenesis

Functional analysis of differentially methylated genes identified 22 Biological Process (BP), 5 Molecular Function (MF), 2 Cellular Component (CC) Gene Ontology (GO) terms and 3 KEGG pathways as significantly enriched (Tables 1 and S4 for the list of annotated genes). Overall, 19 out of 22 GO_BP enriched terms were involved in the development/morphogenesis of anatomical structures, including 10 terms directly linked to embryogenesis (*e*.*g*. proximal/distal pattern formation, embryonic forelimb/hindlimb morphogenesis, spinal cord association neuron differentiation, embryonic digestive tract morphogenesis, anterior/posterior axis specification) and 9 related to specific tissues development (*e*.*g*. muscle organ development, ureter development, neuron differentiation, positive regulation of chondrocyte differentiation). All these terms were mainly represented by genes playing key role in regulating organogenesis (SHH, BMPs, GREM1), body patterning (HOX gene family) and tissues differentiation (FGFR2, FGF18, SOX9).

**Table 1:** Significantly enriched GO terms and KEGG pathways.

Category	ID	Term	Bonferroni-adjusted pvalue
GO_BP	GO:0009954	proximal/distal pattern formation	1.1E-09
	GO:0048665	neuron fate specification	2.8E-08
	GO:0035115	embryonic forelimb morphogenesis	2.1E-07
	GO:0009952	anterior/posterior pattern specification	5.9E-07
	GO:0021522	spinal cord motor neuron differentiation	8.0E-05
	GO:0072189	ureter development	8.2E-05
	GO:0001764	neuron migration	9.8E-05
	GO:0045944	positive regulation of transcription from RNA polymerase II promoter	1.3E-04
	GO:0045666	positive regulation of neuron differentiation	6.7E-04
	GO:0045665	negative regulation of neuron differentiation	1.1E-03
	GO:0007267	cell-cell signaling	1.1E-03
	GO:0035116	embryonic hindlimb morphogenesis	3.2E-03
	GO:0001759	organ induction	5.3E-03
	GO:0003148	outflow tract septum morphogenesis	9.6E-03
	GO:0002053	positive regulation of mesenchymal cell proliferation	1.5E-02
	GO:0007517	muscle organ development	2.2E-02
	GO:0009948	anterior/posterior axis specification	2.9E-02
	GO:0048557	embryonic digestive tract morphogenesis	3.2E-02
	GO:0048664	neuron fate determination	3.2E-02
	GO:0021527	spinal cord association neuron differentiation	3.2E-02
	GO:0032332	positive regulation of chondrocyte differentiation	3.2E-02
	GO:0048701	embryonic cranial skeleton morphogenesis	3.9E-02
GO_MF	GO:0043565	sequence-specific DNA binding	1.6E-10
	GO:0001077	transcriptional activator activity, RNA polymerase II core promoter proximal region sequence-specific binding	2.2E-10
	GO:0000978	RNA polymerase II core promoter proximal region sequence-specific DNA binding	1.1E-06
	GO:0003682	chromatin binding	1.3E-06
	GO:0000977	RNA polymerase II regulatory region sequence-specific DNA binding	2.1E-02
GO_CC	GO:0005667	transcription factor complex	9.0E-06
	GO:0005634	nucleus	6.6E-03
KEGG	5200	Pathways in cancer	3.2E-04
	4080	Neuroactive ligand-receptor interaction	2.3E-03
	4950	Maturity onset diabetes of the young	3.2E-03

**Figure 2:**
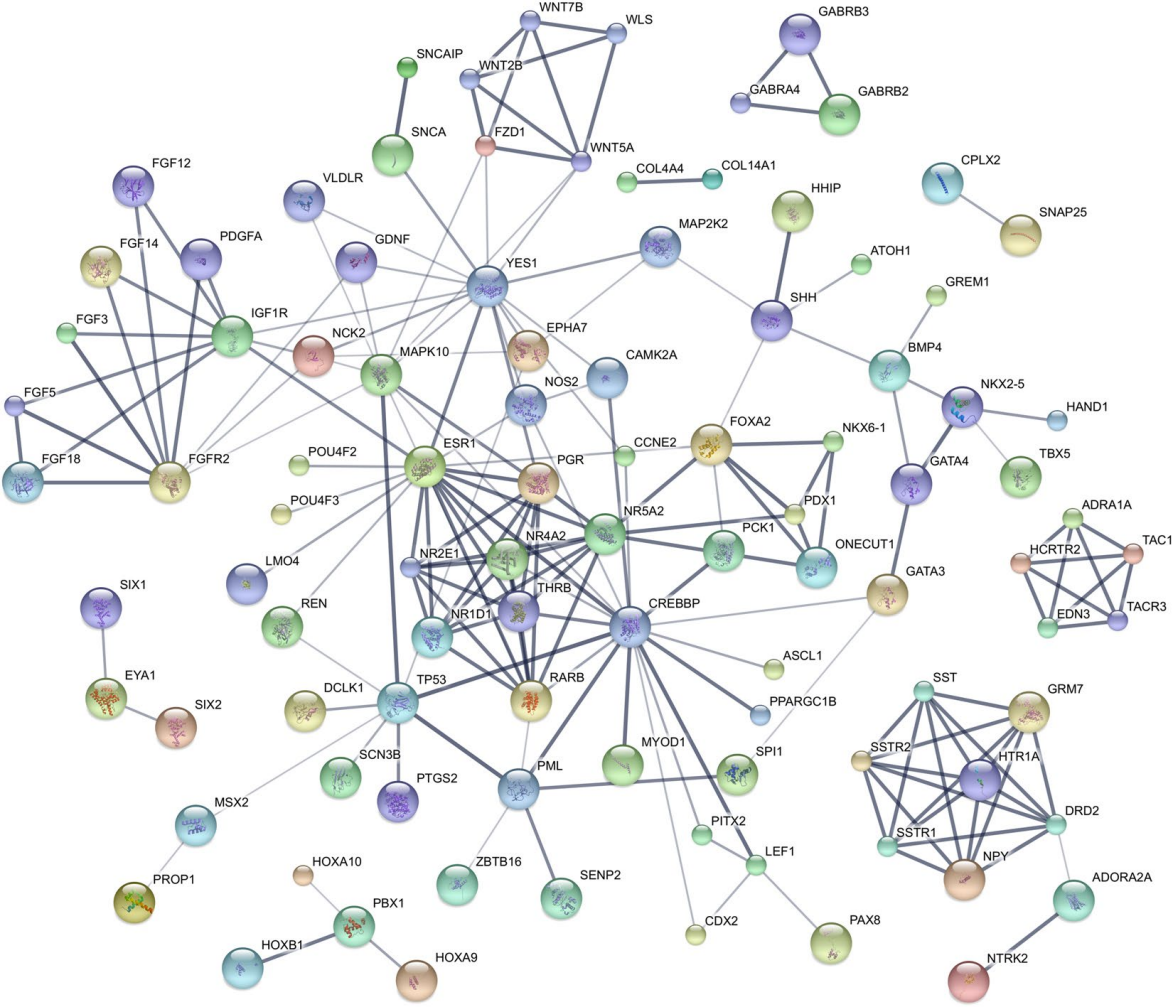
PPI sub-networks from differentially methylated genes belonging to the enriched GO terms and KEGG pathways. The thickness of network edges correlates with the confidential score provided by STRING database: the thicker is the edge, the higher is the confidence score of the interaction.

All GO_MF and GO_CC enriched terms were involved in transcription regulation, as well as one of the enriched GO_BP terms (i.e. positive regulation of transcription from RNA polymerase II promoter). These terms were represented by several transcription factors belonging to different families such as HOX, AP-2, IRX and Zinc-finger transcription factors. Among the 3 significantly enriched KEGG pathways, “Pathways in cancer” (KEGG ID 05200) showed highest significance (Bonferroni-adjusted p-value ≤ 0.001) and included 31 differentially methylated genes (Table S4) involved in key mechanisms such as control of apoptosis (p53, HRK), proliferation (PDGFA, FGFs, RARB, SHH, CCNE1, WNT, SHH) and angiogenesis (NOS2, SLC2A1). The 209 differentially methylated genes belonging to at least one enriched GO_BP, GO_MF term or KEGG pathway (Table S4) were mapped to the protein-protein interaction (PPI) annotations from STRING database^16^: 181 genes shared at least one interaction, 98 of them either experimentally validated or database-curated (Fig. 2).

GSEA confirmed these results with highly enriched GeneSets related to tissue development and morphogenesis (Table S5). Focusing on lymphoid-specific gene expression signatures collected in Staudt’s SignatureDB (https://lymphochip.nih.gov/signaturedb/)^17^, 61 gene sets were found significantly enriched (FDR < 25%), within those 46 showed NOM p-value < 0.05. In particular, two gene signatures specific for DLBCL^18^ and predictive for survival outcome (STROMAL-1 and STROMAL-2_DLBCL_SURVIVAL_PREDICTOR) resulted significant (Table S5).

### Associations between methylation levels and clinical features in cDLBCL

The associations between tumor methylation levels and clinical features (Supplementary File 1 - Table S1) were investigated through statistical pairwise comparisons. For each comparison, samples were grouped as follows: (i) stage (III-IV *vs*. V); (ii) substage (a *vs*. b); (iii) extra-nodal sites infiltration (yes *vs*. no); (iv) treatment (CH *vs*. CH + VAX); v) steroid administration before diagnosis (yes *vs*. no); (vi) relapse (yes *vs*. no OR before the end of therapy *vs*. after the end of therapy + no relapse). Mann–Whitney Wilcoxon test did not detect any association, whereas F-test identified 87 sequences showing a significant differential variability in at least one clinical factor (Table S6). In particular, 4 genes (U6, GRB10, CCDC73, ZFAT) and one miRNA (ENSCAFT00000040944) showed increased methylation variability in dogs relapsing before the end of the therapy whereas 7 genes (OBSCN, THSD1, RNFT2, C6orf201, GRB10, CEP170B, ETV6) were associated to bone marrow infiltration. Only 3 CpG regions were found significant combining the clinico-pathological features by multivariate linear regression model. These were related to exonic regions of EIF2D (stage, LSS) and RNASEH1 (age, therapy and TTP), and the intronic regions of KCNAB2 (age, stage, substage, therapy and TTP). Interestingly, among the 7,526 sequences characterized by a nominal p-value ≤ 0.01 (Table S7), most of the 20 genes associated to a worse clinical behavior (i.e. relapse, TTP and LSS) are involved in regulation of apoptosis and cell cycle (CCND2, SMG7, BCL2L1, BAG1).

### DNA methylation identifies three DLBCL subgroups with different overall survival

**Figure 3:**
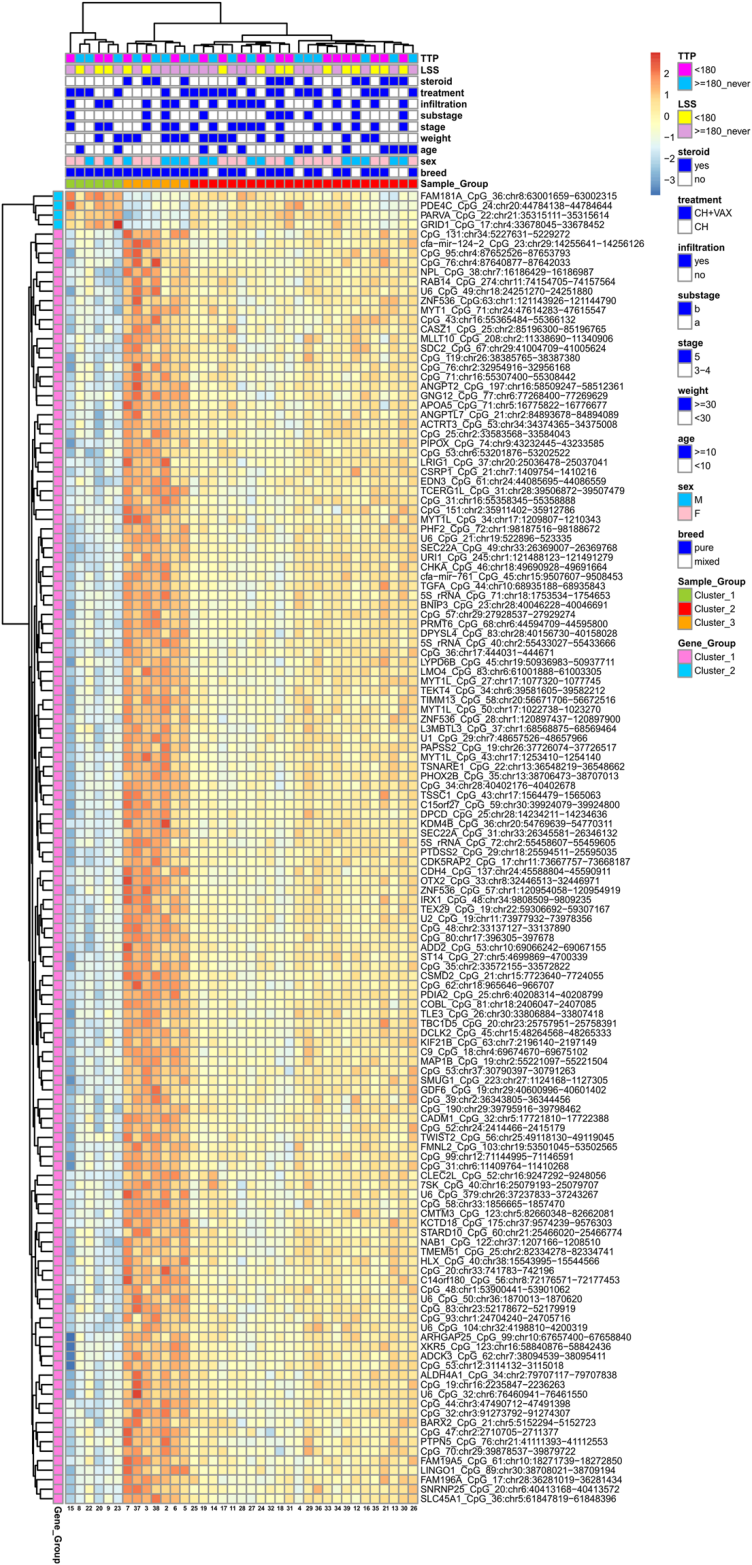
Heatmap of the 138 CpG sequences highly correlated with the first principal component. Methylation levels were centered and scaled by sequence.

**Figure 4:**
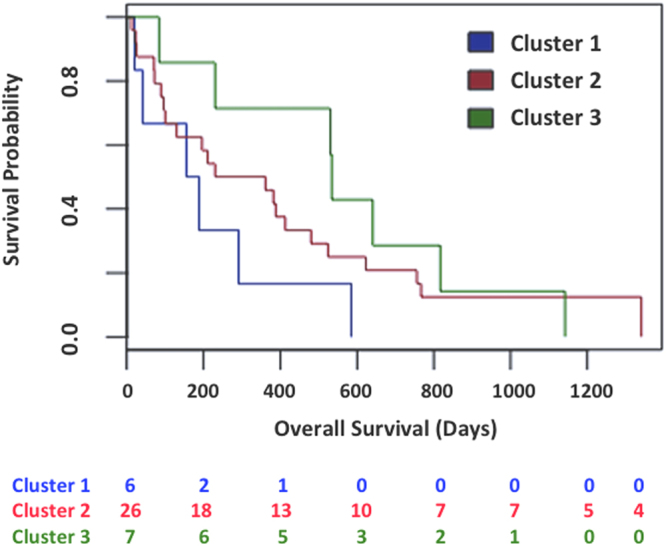
Survival outcomes in patient cohort. Kaplan-Maier curves for LSS according to cDLBCL subgroups defined by PCA and hierarchical clustering on methylation patterns.

Beyond the classification provided by the available clinical factors, we investigated whether methylation profiles were able to provide a different stratification of the cDLBCL samples. The principal component analysis (PCA) explained nearly 25% of the variations in the methylation profiles across cDLBCLs with the first two components (PCs). In total, 138 CpG sequences resulted significantly correlated to the first PC, while no sequences were selected for the second PC. Hierarchical clustering (HCL) on the methylation levels of these CpGs (Fig. 3) identified 3 different subgroups that apparently did not reflect any statistically significant association with the clinical features listed in Table S1. Interestingly, dogs in Cluster#3 showed a higher median LSS (534 days) than Cluster#2 and Cluster#1 (296 and 172 days, respectively). Moreover, Kaplan-Meier curves for LSS confirmed a significant difference in terms of survival between Cluster#3 and Cluster#1 (p-value = 0.02, Fig. 4). Finally, clustering across the 138 selected sequences identified two distinct groups of genes (Fig. 3). The former, including 4 CpG sequences associated to FAM181A, PDE4C, PARVA and GRID1, showed higher methylation levels in Cluster#1 compared with Cluster#2 and #3. Conversely, the latter group, including all the other CpGs, showed an opposite behavior.

### The magnitude of methylation disruption reveals prognostic relevance

The stratification of cDLBCLs considering degree and direction of relative methylation difference between cDLBCL and the mean of control lymph nodes, defined as methylation disruption (see Methods), shows how common hyper- and hypo-methylation events among samples are able to identify distinct cDLBCL subgroups according to methylation variability profiles (MVPs, details in Methods and Supplementary File 1). The first two PCs from PCA on methylation changes explained more than 80% of the variation (Supplementary File 1 - Figure S9). HCL based on the MVPs of CpGs correlating to the first PC identified 3 different cDLBCL subgroups, where the previously identified Cluster#3 was confirmed also by MVPs analysis (Cluster#C in Figure S9).

Focusing on groups of cDLBCLs characterized by methylation disruption driven by the most variable hyper- and hypo-methylation events, consensus HCL was performed on subsets of sequences ranging between 250 and 20,000, sorted by decreasing median absolute deviations (MADs) on MVPs. Interestingly, up to the first 2,000 sequences with the highest MADs the clustering was able to identify a specific subgroup of 6 cDLBCLs characterized by the highest variability of differential methylation changes between cDLBCLs and control lymph nodes (Supplementary File 1 - Figure S10); 5 of these dogs showed short-term LSS (LSS < 180 days, p-value < 0.03, Fisher’s Exact Test).

### Technical and functional validation of microarray results

A technical validation of microarray platform by methylation-specific PCR was performed on 5 differentially methylated genes (FGFR2, HOXD10, RASAL3, CYP1B1 and ITIH5) involved in key biological pathways of cancer development (*e*.*g*. MAP/ERK, Homeobox signaling and FGF signaling pathways). The methylation levels were assessed in 13 cDLBCLs and 5 control lymph nodes. The promoters of the tested genes resulted hyper-methylated and a statistical significance for HOXD10, RASAL3, CYP1B1 and ITIH5 was found (p < 0.01), thus confirming the reliability of the microarray platform (details in Supplementary File 1).

A functional validation of microarray data was also performed selecting 3 hypermethylated genes (CADM1, CDH11 and ABCB1). In this respect, the mRNA restoration after the treatment of a canine B-cell lymphoma cell line (CLBL1)^19^ with two hypomethylating agents (azacytidine and decitabine) was evaluated through quantitative Real Time PCR. The three transcripts resulted highly expressed in control lymph nodes and scarcely expressed in CLBL1 cells. After azacytidine treatment a significantly restoration of expression for the three genes was found, conversely decitabine affected only ABCB1 expression (Supplementary File 1).

## Discussion

Origin of B-cell lymphoma in dog results from inherited mutations in the germ line or changes in DNA sequences during life^20^. Recently, extensive gene expression profiling identified molecular signatures of cDLBCL and defined two distinct subgroups with prognostic significance^2, 3^. However, the biology of this tumor is still not entirely explained by genomic events and transcriptional programs, and much less is known about epigenetic changes^11^. Therefore, genome-wide DNA methylome has been investigated in this study using for the first time a canine DNA CpG microarray. Results revealed that cDLBCLs are characterized by a widespread aberrant methylation affecting 1,194 regions, corresponding to 823 genes. The hyper-methylated sequences were enriched in upstream (≤10 kb) or promoter regions, while the hypo-methylated sequences were preferentially located in gene bodies and downstream regions. This was quite expected considering that CpGIs are highly susceptible to DNA methyltransferases in cancer, determining gene silencing. Conversely, CpG-poor regions undergo to a global decrease of genomic DNA methylation affecting genome stability, transcriptional elongation, and RNA splicing^9, 21–23^.

In order to indirectly confirm the relationship between methylation and transcription in cDLBCL, we integrated our data with the cDLBCL gene expression dataset published by Mudaliar *et al*.^2^. A total of 107 significant genes were found in common between the two platforms and 98% of the hypermethylated upstream/promoter regions resulted downregulated. The same was observed for hyper-methylated CpGIs in gene bodies. By contrast, hyper-methylation of the downstream regions and CpGIs hypo-methylation did not show any consistent relationship with gene expression. Hypo-methylated CpGs-poor CDS regions represented the only exception, being downregulated in tumors.

The biological processes significantly enriched in cDLBCLs appear to be strongly related to embryonic development, tissue morphogenesis and cellular differentiation. DMRs included genes encoding proteins with key roles in development and cell fate determination in all types of cells, such as HOX, BMP, WNT, and SOX. Interestingly, inappropriate or deregulated expression of HOX genes has been implicated in several human cancers and associated to promoter methylation^24–26^. Furthermore, the expression of HOX genes during maturation of hematopoietic cells results tightly regulated and modifications of this mechanism seem to contribute to neoplastic transformation. The hyper-methylation of several members of HOX gene family in our study suggests a possible contribution to cDLBCL pathogenesis and points to the importance of certain signaling pathways across human and dog.

Wnt pathway is one of the “usual suspects” in cancer biology. The cellular processes modulated by Wnts range from stem cell self-renewal to cell motility, and are mediated by transcriptional activation as well as through direct effects on cytoplasmic targets^27^. In the present study, several components of this pathway (i.e. WNT2B, WNT5A, WNT7B, FZD1 and LEF1) were found hyper-methylated in cDLBCL. This signaling cascade is multifaceted and somehow enigmatic since it has been widely reported that some members can play tumor-promoting or suppressing role depending on the cell type or availability of key receptors^28, 29^. An emblematic example is given by Wnt5a, reported overexpressed in several human cancers^27, 29^, that in the presence of specific FZ isoforms, could promote tumor growth by activating the cancer-promoting canonical Wnt signaling pathway. However, in hematological malignancies, including B-cell lymphoma, WNT5A acts as a tumor suppressor able to antagonize the WNT/β-catenin signaling^30–32^ and it is found silenced by tumor-specific methylation. Hyper-methylation of Wnt5a promoter was observed in the present study, supporting its role in inhibiting B-cell proliferation, while the significance of FZD1 and LEF1 epigenetic silencing remains unclear. To date, both genes were reported activated in several cancers and associated to chemoresistance and poor prognosis^33, 34^.

BMPs frequently inhibit cell differentiation and proliferation and are involved in cancerogenesis in different manners. Indeed, there are controversial *in vitro* and *in vivo* studies regarding the role of BMPs in promoting tumorigenesis and metastasis^35^ and showing biological behavior associated with cancer origin^36^. However, several studies reported that BMPs can influence the hematopoietic system and regulate development of hematopoietic stem cells. Interestingly, B- and T-cell lymphopoiesis is inhibited by inducing the activation of Smad 1/5/8^37^. In the present study, three BMPs members belonging to the Transforming growth factor beta (TGF-β) superfamily, namely BMP3, BMP4 and BMP7, were found hyper-methylated in cDLBCL, supporting the role of BMP families as tumor suppressor genes. BMP7 was demonstrated to be methylated both in human DLBCL and follicular lymphoma indicating its physiological relevance^38^.

In our study, we identified a high proportion of Polycomb Group (PcG)-target genes (243/823, 29.5%) differentially methylated. The genes included WNTs, Hedgehog, BMPs, PAXs, FGFs, and FOX factors. Polycomb group (PcG) proteins represent a global silencing system involved in development control and they are able to regulate the transition from proliferation to differentiation, contributing to stem-cell maintenance, and inhibit inappropriate activation of differentiation programs. Recent studies have provided evidence that PcG-target genes are frequently hyper-methylated in several tumors, including lymphoma, and our findings confirm the same trend for cDLBCL. As proposed by Martín-Subero *et al*.^39^, this scenario allows a double interpretation. The most consistent relies on the effect of PcG-target genes aberrant methylation in tumor precursor cells with stem cell–like features initially conferring a growth advantage and abnormal proliferation. In turn, this uncontrolled expansion and the consequent genome instability would promote the gain of further oncogenic mutations (i.e. chromosomal aberration), which foster malignancy progression^22, 40,41]^. This concept supports the “cancer stem cell theory” which states that primordial cancer-initiating cells, exhibiting self-renewal capacity and multilineage potential, may expand and form the biological origin for the rest of the tumor^42, 43^. The second hypothesis relies on the capacity of the neoplastic cells to secondary acquire a stem cell-like epigenetic pattern through gene deregulation caused by chromosomal aberrations^39^. The mechanisms regulating the interplay between DNA promoter methylation and PcG-driven silencing, if any, are still unknown. DNA methylation and histone methylation (i.e. H3K27me3) directed by the PcG family protein EZH2 are mutually exclusive in normal cells, but this relationship seems not maintained in cDLBCL^22, 40^. In human, mutations in the SET domain of EZH2 are associated to aberrant epigenetic events both in DLBCL and follicular lymphoma^44, 45^, whereas this is the first evidence supporting a role of PcG proteins in dog and further investigation are necessary to study the mechanism of EZH2 in cDLBCL.

In human DLBCL, intra-tumor and inter-patient variability in promoter DNA methylation, as well as specific methylation states have been reported to be associated to tumor clinical behaviour^46–48^. Here, the F-test was applied for our data and the analysis yielded 4 genes (U6, GRB10, CCDC73 and ZFAT) with the highest ability to predict survival. Both GRB10 and ZFAT are known to play a role in regulating hematopoietic stem cell self-renewal and haematopoiesis and several studies revealed an association with cancer progression^49–51^. However, their contribution in B-cell lymphoma remains poorly understood. One limitation of our study is the number of cases that might affect the statistical power of testing differential methylation for the clinical features. Further studies with a higher caseload are needed to assess the associations between specific gene methylation and clinical behaviour.

A second aim of this study was to explore whether epigenetic profiles might help to classify cDLBCLs into biologically relevant subgroups. Analysis of methylation levels identified 3 potential subgroups described by 138 CpG sequences which mainly contributed to methylome-wide variations among the cDLBCLs and characterized by biological pathways that are mainly related to nervous system development, cell differentiation and morphogenesis (data not shown). A clear correspondence with the canine ABC and GCB-like DLBCLs, characterized by a differential expression of NF-κB and B-cell receptor pathways, was not established^3^. A comparative approach was attempted by considering the canine-specific geneset, reported by Richards *et al*.^3^, whose expression was able to distinguish cBCL into “ABC-like” and “CCB-like” subtypes. Out of 787 genes (1,180 probes), 597 (1,464 features) were represented in the dog CH3 microarray and the corresponding methylation levels were employed as dataset to perform HCL of cDLBCLs. No distinct separation into two subgroups was achieved; the same evidence was obtained when limiting the dataset to those regions (n = 17) already found differentially methylated between cDLBCL and control lymph nodes (data not shown).

Interestingly, one of the cDLBCL subgroups defined by our methylation profiles (Cluster#3) is associated to long-term survival with respect to the other samples. Comparable results were obtained by stratifying cDLBCLs considering the magnitude of methylation changes with respect to control lymph nodes. This latter analysis reproduces the methodological approach described in Chambwe *et al*., where 6 subgroups of DLBCL in human with prognostic relevance were identified, “refining” the ABC- and GCB-DLBCL classification^46^. The main advantage of this approach is exploiting the methylation profiles of control samples as a reference in order to perform PCA and clustering focusing on the hyper- and hypo-methylation events in each sample. In the present study, 3 cDLBCL subgroups with different magnitude of DNA methylation changes were identified, confirming the Cluster#3 characterized by long-term survival (Cluster#C). Since many sequences were characterized by low differential methylation levels, which might confound the detection of small cDLBCL subgroups defined by a limited number of sequences showing high variations of methylation disruption, a consensus HCL was performed on subsets of sequences sorted by decreasing MADs of the MVPs. Interestingly, a cluster of 6 dogs based on about 2,000 sequences characterized by the highest methylation variability changes was significantly associated to poor prognosis (LSS < 180 days, p < 0.03). The consensus clustering showed that 6 dogs characterized by poor prognosis robustly clustered together and not with the other samples of the two other clusters by performing HCL several times on subset of samples (Figure S10C). In addition, methylation changes observed in these dogs were characterized by a higher number of hyper-methylation events compared to the other samples, as shown by the average of density plots of their methylation changes (Figure S10A) and the highest Methylation Variability Scores (Figure S10B) defined in Chambwe *et al*.^46^, (i.e. quantitative measures reflecting the magnitude of methylation disruption). This finding supports the hypothesis that the progressive accumulation of aberrant epigenetic changes might confer aggressiveness to the tumor. Future studies with a higher number of cases will be definitely necessary to better assess the contribution of epigenetic profiles in both biological and clinical stratification of cDLBCL. In this context, investigating the level of methylation variability of cDLBCL might be a starting point to highlight the contribution of DNA methylation in the clonal evolution of this tumor^6^.

In conclusion, we profiled genome-wide DNA methylation in cDLBCLs using DNA methylation array and we technically and functionally validated our results by methylation-specific PCR and the treatment of a canine B-cell lymphoma cell line with hypomethylating agents, providing insights on epigenetic switching and heterogeneity of this tumor. Findings collected, herein, suggest that cDLBCL aberrantly has tumorigenic and stem cell-like signatures, highlighting some methylation-based cDLBCL subgroups showing prognostic relevance. In future, new brand high-resolution techniques will be required to better define the contribution of methylation in cDLBCL associated with functional studies of the aberrant methylated genes and the identification of putative tumor biomarkers to predict the clinical outcome.

## Methods

### Dogs and samples

The study cohort included 40 dogs affected by newly diagnosed, multicentric DLBCL that underwent complete and standardized staging work-up and that were treated with chemotherapy or chemo-immunotherapy^52^. The diagnosis of DLBCL was obtained by histopathological and immunohistochemical analysis (CD20 and CD79) of one enlarged peripheral lymph node surgically removed at initial presentation. A portion of the tumor was preserved frozen in RNAlater® solution (Life Technologies, Carlsbad, CA) under sterile conditions. Medical records of all dogs were reviewed to obtain relevant clinical information, including signalment, breed, sex, age, hematological and biochemical abnormalities, clinical stage, substage and treatment (Supplementary File 1- Table S1). Time to progression (TTP) was measured as the interval between initiation of treatment and progressive disease (PD). Dogs not experiencing PD at the end of the study or dogs lost to follow-up before PD were censored for TTP analysis. Lymphoma-specific survival (LSS) was measured as the interval between initiation of treatment and lymphoma-related death. 8 dogs with no cancer disease and defined clinically healthy by a complete blood exam and physical examination underwent lymphadenectomy and lymph nodes were used as controls. Samples stored in RNA-later were analyzed to selectively extract DNA from regions compatible with lymphoid follicles.

The study was approved by Committee of the University of Padova (CPDA148778/14 - protocol 20086MSFH3) and a mandatory written consent from all dog’s owners was obtained. All the experiments were performed in accordance with relevant guidelines and regulations.

### Dog CH3 microarray design

In order to assess methylation profiles of dogs affected by DLBCL, a canine CpG microarray platform was developed (GEO accession: GPL23069). Probe design was carried out by the Agilent bioinformatic support team using proprietary prediction algorithms to locate CpG Islands on *C*. *familiaris* draft genome as deposited on Ensembl database (CanFam 3.1) and to design high quality oligo-probes. Microarray probes were selected in order to provide the highest possible coverage of dog genome. CDS regions and CpG islands were given top priority. Chromosome X was excluded from analysis and probe design. A total of 170,000 probes (60mers, sense orientation) were designed on both CpG and CDS regions. In details, 102,000 probes were designed targeting a total of 36,807 CpG regions while 68,000 probes were directed against 672 CDS; average base pare tiling was 90 bp. Microarray probes were synthesized *in situ* using the Agilent non-contact ink-jet technology with a 4 × 180 K format. Each array included Agilent’s default positive and negative controls.

### Sample processing and data normalization

Detailed description of sample processing, data quality assessment and normalization is reported in Supplementary File 1. Briefly, for both cDLBCLs and control lymph nodes, DNA methylation was measured by two-color competitive hybridization between the methylated fraction and a not-enriched aliquot of the same DNA. Loess normalization was applied to correct for the Cy3/Cy5 dye bias for each dye. After quality control (QC) of the resulting log2 ratios, between-samples Quantile normalization was then applied to the QC-passed arrays (37 cDLBCLs and 7 controls).

### Data Analysis

Data analyses were performed using R statistical computing software (http://www.r-project.org). Details are reported in Supplementary File 1. The median of the probe signal was calculated to retrieve the methylation levels of the target 672 CDS and 36,807 CpG regions. DMRs between cDLBCLs and lymph nodes were identified by Mann–Whitney Wilcoxon test, filtering out sequences showing enriched methylation in less than 25% of the two groups (i.e. cDLBCLs and control lymph nodes). Both Mann–Whitney Wilcoxon and F-test for differential variability were then applied to study associations between gene methylation levels and clinical features. Possible combinations of multiple clinical features were also investigated by multivariate linear regression model. For all the statistical tests, Bonferroni-adjusted p-values < 0.01 were considered significant. Functional characterization of DMRs was performed by different state-of-the-art enrichment approaches as topGO and Gene Set Enrichment Analysis (GSEA).

The ability of methylation profiles in defining new tumor stratifications was first investigated by applying PCA. Target sequences showing high correlation (i.e. above 0.85) with the main principal components (PCs) were selected by using one-way analysis of variance^53^. Focusing on these sequences, HCL using Euclidean distance and Ward linkage was applied to identify cDLBCLs subgroups characterized by different methylation profiles and clusters of CpG/CDS regions characterized by a specific methylation pattern across cDLBCLs.

PCA and HCL were applied to MVPs, representing the density functions of the differential methylation levels between each cDLBCL and the median methylation level calculated across control lymph nodes. Clustering was performed according to a distance defined by the area bounded by the MVP-based density curves of sample pairs (Supplementary File 1). Moreover, consensus clustering on subsets of sequences sorted by decreasing MAD was applied to stratify according to sequences characterized by highly variable methylation disruption across cDLBCL samples. To evaluate the robustness of the results, the consensus clustering provides quantitative evidence for determining the number and membership of possible clusters by randomly subsampling 1,000 times the samples. Clustering performance from subsampling are summarized by a consensus matrix with values ranging between 0 (never clustered together) and 1 (always clustered together). In addition, each cDLBCL sample was characterized by a Methylation Variability Score (MVS) representing the difference of the area under the density curves between the MVP of each cDLBCL sample and the median MVP from the controls: the higher the MVS, the greater the methylation disruption^46^. Further details on the analysis of methylation disruption and the consensus clustering are reported in Supplementary File 1. Finally, Fisher’s exact test was performed on the whole CpG-probe set β values treated as categorical data to identify the differentially methylated probes. The probes were classified as “methylated” (β value ≥ 0.5) or “unmethylated” (β value < 0.5) (for details see Table S3).

## Electronic supplementary material

Supplementary file 1

Table S2

Table S3

Table S4

Table S5

Table S6

Table S7
