# Supplementary material for: DNA methylation profiling reveals common signatures of tumorigenesis and defines epigenetic prognostic subtypes of canine Diffuse Large B-cell Lymphoma
Source: Sci Rep. 2017 Sep 14;7:11591. doi: 10.1038/s41598-017-11724-w (PMC5599585; doi:10.1038/s41598-017-11724-w)
Supplement: Supplementary file 1 [file 41598_2017_11724_MOESM1_ESM.pdf]

SUPPLEMENTAL INFORMATION

DNA methylation profiling reveals common signatures of tumorigenesis and defines epigenetic prognostic subtypes of canine Diffuse Large B-cell Lymphoma

Serena Ferraresso, Arianna Aricò, Tiziana Sanavia, Silvia Da Ros, Massimo Milan, Luciano Cascione, Stefano Comazzi, Valeria Martini, Mery Giantin, Barbara Di Camillo, Sandro Mazzariol, Diana Giannuzzi, Laura Marconato, Luca Aresu

Table of Contents

**Supplemental Materials and Methods**	**2**
cDLBCL cohort	2
DNA extraction and sonication	3
Enrichment of methylated double-stranded DNA	3
Sample labeling and hybridization	3
Data Quality Control and Preprocessing	3
Details on statistical analyses	4
Annotation and functional analysis	5
Bisulfite conversion	6
Methylation specific PCR (MSP)	6
Gene expression analysis of CLBL1 cells treated with hypomethylating agents	7
**Supplemental Results**	**9**
Data preprocessing	9
Microarray data technical validation	9
Microarray data functional validation	11
Supplemental References	12
Supplemental Figures	13
Supplemental Tables	15

SUPPLEMENTAL MATERIAL AND METHODS

cDLBCL cohort

Clinical features of dogs affected by DLBCL are reported in Table S1.

Table S1. Clinical data of DLBCL dogs included in the study

Dog Number	Age (y)	Sex	Stage	Sub-stage	Extranodal site infiltration	Treatment	Pre-Pred	Relapse pre/post end of therapy
**1***	13	M	5	b	BM 13,5%	CH+VAX	yes	post
**2**	5	M	5	b	BM 12,7%, lung	CH+VAX	no	post
**3**	8	F	5	b	lung	CH	yes	pre
**4**	12	F	3	a	no	CH+VAX	no	never
**5**	5	M	3	a	no	CH+VAX	yes	post
**6**	3	M	5	a	BM 4,3%	CH	no	pre
**7**	7	M	4	a	no	CH	yes	pre
**8**	8	F	4	a	no	CH+VAX	no	never
**9**	3	M	5	a	PB 10%	CH	no	pre
**10***	13	F	5	b	BM 7,2%	CH	no	pre
**11**	8	F	5	a	BM 3,1%	CH+VAX	no	post
**12**	6	M	5	b	BM 47,4%	CH	yes	pre
**13**	10	M	3	a	no	CH	yes	post
**14**	6	M	5	a	skin	CH+VAX	no	post
**15**	9	F	5	b	lung	CH+VAX	no	pre
**16**	5	M	4	a	no	CH+VAX	yes	post
**17**	10	F	4	a	no	CH+VAX	no	pre
**18**	5	F	5	b	BM 5,3%	CH+VAX	yes	pre
**19**	10	M	4	b	no	CH+VAX	no	pre
**20**	9	F	5	a	BM 55,2%	CH	no	pre
**21**	13	F	4	a	no	CH+VAX	yes	pre
**22**	5	M	4	a	no	CH+VAX	no	post
**23**	10	F	4	a	no	CH+VAX	no	never
**24**	5	M	5	a	BM 4,7%	CH	no	pre
**25**	6	F	5	a	BM 5,7%	CH+VAX	no	post
**26**	10	F	4	a	no	CH+VAX	no	post
**27**	5	M	5	a	BM 6,6%	CH+VAX	no	post
**28**	10	F	5	a	BM 14,9%	CH	yes	pre
**29**	11	F	4	b	no	CH+VAX	yes	post
**30**	10	M	5	b	lung	CH	yes	pre
**31**	6	M	4	b	no	CH	yes	pre
**32**	4	F	4	b	no	CH+VAX	yes	post
**33**	12	F	4	a	no	CH	no	pre
**34**	4	F	5	a	BM 11,2%	CH+VAX	no	pre
**35**	8	F	5	a	BM 5,1%	CH+VAX	no	pre
**36**	4	F	5	a	BM 5%	CH+VAX	no	never
**37**	5	F	4	a	no	CH	no	never
**38**	5	F	4	a	no	CH	yes	post
**39**	10	M	4	a	no	CH	no	pre
**40***	10	M	5	a	BM 5%	CH	yes	pre

M=male, **F**= female, **Pre-pred**= steroid administration before diagnosis, **BM**=Bone Marrow Cells; **PB**=Peripheral Blood cells; **CH**=chemio; **VAX**=vaccine. **(*)**: Sample excluded from statistical analyses

DNA extraction and sonication

Genomic DNA was extracted from lymph nodes using the DNeasy Blood & Tissue Kit (QIAGEN, CA, USA) according to the manufacturer's instructions. DNA concentration and quality were measured by Qubit fluorometer (Life Technologies, CA, USA) and by Agarose gel electrophoresis. An amount of 5 µg of extracted genomic DNA was fragmented by sonication, using Covaris S2 (Covaris, MA, USA), to obtain a fragmented DNA that ranges from 200 to 700 bp in size, diluted in 130 µl. Covaris settings were the following: n° cycles= 2, cycle duration= 60 sec, duty cycle= 10%, cycle/burst= 200 and intensity= 5, with a bath temperature of 5±1°C. An aliquot of 20 µl (total 700-800 ng) was used as reference DNA, not enriched of methylated double-stranded DNA.

Enrichment of methylated double-stranded DNA

The remaining aliquot (110 µl, ~4 µg) of fragmented DNA was enriched of methylated double-stranded DNA by using MethylMiner™ Methylated DNA Enrichment Kit (Life Technologies), following the manufacturer's recommendations. MethylMiner™ uses a biotinylated recombinant fragment of the human MBD2 protein to enrich for fragments of methylated DNA. The methylated fraction of genomic DNA thus obtained was employed for methylation analysis through canine methylation microarray.

Sample labeling and hybridization

Enriched-fraction and total gDNA (reference) obtained from 48 lymph node samples (40 cDLBCLs and 8 control dogs) were labelled independently with cyanine 5-deoxyuridine triphosphate (dUTP) and cyanine 3-dUTP, respectively. Sample labeling was performed by using SureTag Complete DNA Labeling Kit (Agilent Technologies) following the manufacturer's recommendations. For each sample, equal amount of enriched (Cy5-labeled) and reference (Cy3-labeled) DNAs were co-hybridized to the microarray platform. Arrays were scanned at 3µm resolution using an Agilent G2565CA scanner, and image data were processed using Feature Extraction version 10.7 with CGH-1200-Jun14 protocol (Agilent Technologies).

Data Quality Control and Preprocessing

A total of 58 probes exhibiting signal saturation and one cDLBCL sample not valid according to Methylation Microarray QC metrics (Agilent Technologies) were filtered out. The MedianSignal of the probes was considered for further preprocessing. The ProcessedSignal provided by Agilent Feature Extraction algorithm was not employed since it was characterized by a higher overall variability. Differences on signal variability were observed between Cy3 and Cy5 signals, probably due to the capture/enrichment step performed on DNA of Cy5-labeled samples. To adjust the Cy3/Cy5 dye bias, Loess normalization was applied to each dye using the information between-

array to remove intensity-dependent trends, but without scaling the overall median signal towards zero. Specifically, the Loess curve was estimated keeping the within-array median value calculated across the probes. After dye bias correction, quality assessment of the resulting log2-signal ratios was performed using the arrayQualityMetrics package in Bioconductor (http://www.bioconductor.org). Principal Component Analysis (PCA) was also considered to evaluate anomalies among the samples. Three samples (i.e. Dog#1, Dog#10 and one control dog) failing quality controls on MA plots, box-plots and between-array distances were excluded from the analysis. On the remaining arrays, between-samples Quantile normalization was applied to the corresponding log2-signal ratios. The distribution of the median log2-ratios calculated across samples was characterized by two clear peaks (Figure S1): the first one (log2-ratios > 2) represented hyper-methylated probes, while the second one (0 <log2-ratios <1) represented probes showing a methylation level similar to the reference. The latter was not centered to zero since the MedianSignal included the background noise that may be different between the dyes. Therefore, in order to subtract the background noise and having this peak centered to zero, the overall signal was finally scaled by a factor equal to 0.85. To estimate the peaks, we used the function findPeaks of R package quantmod.

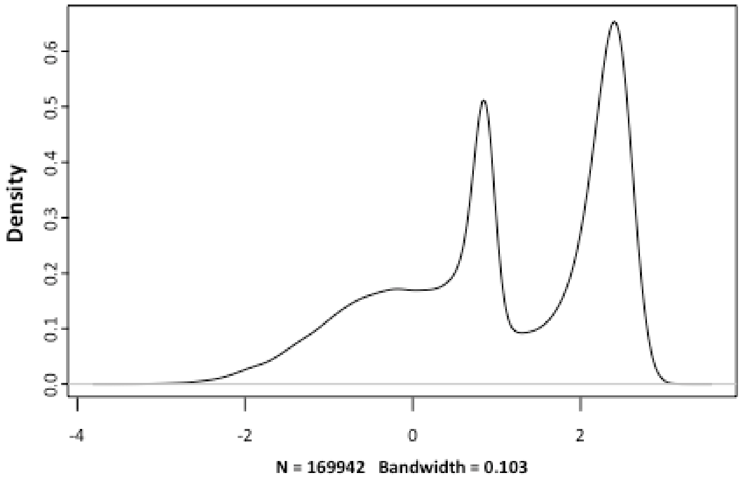

Figure S1. Density plot of the median array calculated from the normalized data.

Details on statistical analyses

F-test: Since cDLBCL is a heterogeneous disease, driven by perturbations of different molecular pathways, and varying from individual to individual, epigenetic instability or the loss of epigenetic control of important genomic domains can lead to increased methylation variability, not always associated to a difference of methylation levels. Recently, it has been found that differential variability between normal and cancer tissues can be very useful for identifying methylation markers of cancer (Hansen et al., 2011). Therefore, for the clinical factors differential variability

was tested using the F-test, one of the most popular approaches for testing the equality of variances.

Multivariate linear regression: Linear combinations of clinical/pathological factors significantly associated to methylation level were investigated through a multivariate linear regression model. Specifically, the factors of the final model were selected with a step-down procedure: all the factors were initially included in the full model considering main effects only, then they were sequentially removed if their removal did not result in a significant change in fitting the data, using F-test.

Analysis of methylation disruption: Starting from methylation data in cDLBCL samples and control lymph nodes a matrix X was defined describing the methylation changes for each sequence j and each cDLBCL sample i as $x_{ij} = y_{ij} - z_j$, which is the methylation difference between the sample i and the median methylation z_j calculated across the 7 control lymph nodes at sequence j. PCA analysis was performed on x_{ij} values as preliminary analysis of the variability in methylation changes. The methylation variability profile for each cDLBCL sample i (MVP) was then defined as the density function $f_i(x)$ across all the regions represented on the array. The function was estimated using the density() function in R with bandwidth parameter 0.1[33].

To define a distance matrix for the clustering, the squared L2-distance between the MVP density functions were calculated for all pairs of patient samples. This distance represents the squared difference in the area under the curve between two samples and is approximated using the Trapezoidal rule (Supplementary Material in Chambwe et al.[33]).

Consensus clustering was then performed on this matrix applying Ward's linkage, using R package ConsensusClusterPlus (Wilkerson et al., 2010). Specifically, HCL was performed 1,000 times on resampled subsets of the cDLBCL samples (using 80% of samples as subset) and evaluated the number of clusters k=2,3……15. We note that the relative change in area under the cumulative distribution functions of the consensus matrix (described in Methods) for each k is maximum at 3, indicating the best separation of the clusters.

Finally, to provide also a quantitative measure of the magnitude of methylation disruption observed in each sample, Methylation Variability Score of cDLBCL sample i was defined as the deviation of each cDLBCL MVP describe by $f_i(x)$ to that of the expected MVP of a control lymph node, described by the mean density function $\overline{g}(x)$[33]:

$$MVS_i = \int [f_i(x) - \overline{g}(x)]^2 dx$$

Annotation and functional analysis

In order to improve the biological interpretation of the significant sequences, CanFam3 annotations from both RefSeq and Ensembl retrieved from UCSC table browser were associated to each sequence. In particular, we first checked whether each sequence overlaps at least one of the

following genomic locations: 5’-UTR, 3’-UTR, exonic, intronic, promoter/upstream (i.e. 2k/10k bases upstream from transcription start site, respectively), downstream (i.e. 2k bases from transcription start site). If the sequence did not overlap any of these locations (i.e. it is an intergenic region), the nearest gene was associated, assuming possible distal regulatory effects on the associated gene.

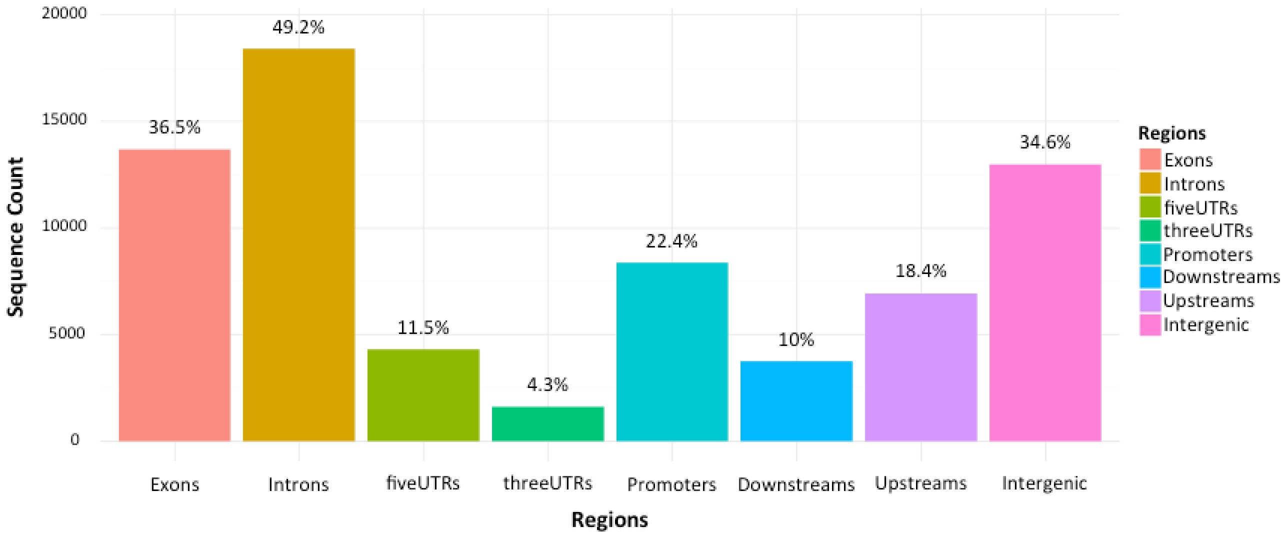

Figure S2. Distribution of target sequences (CpG and CDS) across the dataset. Percentages with respect to the total number of sequences in the chip are reported. The sum of these percentages is not equal to 100% since each sequence can overlap more than one genomic region.

In order to identify enriched genomic locations with respect to the selection of the differentially methylated sequences, Fisher’s Exact test was performed on the number of the selected sequences with respect to the total number of sequences available in the microarray platform.

Finally, the biological terms from Gene Ontology (GO) and KEGG pathways able to significantly characterize the selected sequences were identified by performing an enrichment analysis. topGO R package with default options (Alexa et al., 2006) and Fisher's Exact test were applied on GO terms and KEGG pathways respectively, considering as significant the terms/pathways with adjusted Bonferroni p-value <0.05. Functional annotations were retrieved from R packages org.Cf.eg.db, GO.db and KEGG.db. Focusing on the selected sequences belonging to the enriched terms, the corresponding protein-protein interactions (PPIs) derived from STRING database were considered for further interpretation of the obtained results (Szklarczyk et al., 2015).

Gene set enrichment analysis (GSEA) was performed on the entire dataset using the Gene Set Enrichment Analysis v2.0.13 software (Subramanian et al. 2005) downloaded from the Broad Institute (www.broadinstitute.org/gsea.). GSEA analysis was performed by using gene symbols retrieved by blastx against UniProt database. For enrichment analysis Gene sets were downloaded

from the C2-CP C4-CM and C6 collections in MsigDB v3.1 (Molecular Signature Database). In addition, more specific lymphoid gene sets were retrieved from Staudt's SignatureDB (https://lymphochip.nih.gov/signaturedb/, Shaffer et al. 2006). Pathway Enrichment analysis was performed on each collection independently, T-test metric was employed for gene ranking, and 1,000 permutations were applied for p-value assignment.

Bisulfite conversion

Genomic DNA from 13 cDLBCLs (Dog number:1, 6, 14, 15, 18, 19, 20, 24, 30, 32, 33, 34, 38) and five lymph node samples (Ctrl#1, Ctrl#2, Ctr#4, Ctr#7, Ctrl#8) was quantified using NanoDrop1000 Spectrophotometer (Thermo Scientific). For each sample, 500 ng of genomic DNA were bisulfite treated using the MethylCodeTM Bisulfite Conversion Kit (Invitrogen™, Carlsbad, California) following manufacturer's specifications. Bisulfite-converted DNA was then employed as template for MSP.

Methylation specific PCR (MSP)

A technical validation of microarray platform by methylation-specific PCR (Hernández et al. 2013) was performed on 5 differentially methylated genes (FGFR2, HOXD10, RASAL3, CYP1B1 and ITIH5). On the CpG islands of these genes (Table S2), Methylation-specific primers were designed by means of Methyl Primer Express software (Applied Biosystems, Foster City, CA). For each gene, two primer sets were designed: i) METH primers designed to amplify the DNA if methylated (scenario in which the cytosines in CpG dinucleotides are methylated and are not be bisulfite converted into uracil); ii) NO-METH primers designed to amplify the same DNA if not methylated (scenario in which all cytosines are supposed to be bisulfite converted into uracil).

All the MSPs were carried out using 5 ng of bisulfite-converted gDNA; 600-600 nM primer pair was used for all genes apart from CYP1B1 (300-600nM METH, 600-300nM NO-METH), ITIH5 METH (300-600nM), RASAL3 METH and NO-METH (50-50 nM). Real time amplification was carried out using the Master Mix SYBR® Green PCR Master Mix Applied Biosystems and Stratagene Mx3000P Agilent Technologies. For CYP1B1 and ITIH5 genes the Ct values were acquired at 76 and 74 °C, respectively, to eliminate primer dimer contribution to the amplification plot. Negative controls (with no bisulfite-converted gDNA or water as template) were run in every plate for each assay. The quantification of methylation level for each target gene was carried out by calculating the ratio of methylated to unmethylated primers pairs as ΔCT (=CT_Meth – CT_NoMeth) as described by Zeschnigk et al. (2004).

Statistical analyses were performed using a commercially available statistical software program (SPSS v20.0). Data were analysed using a non-parametric statistical method because of the limited

number of cases. Sample methylation levels, were evaluated for significant differences between controls and cDLBCLs using the Mann-Whitney test.

Table S2. Primer pairs employed for MSP

Gene		Methylation primer 5'-3'	No Methylation primer 5'-3'
**FGFR2**	Forward<br>Reverse	GTTATACGGGGGCGTTGAC<br>GCGAAAACCAAATACCGAATACG	TGTGGTTATATGGGGGTGTTGAT<br>ACTCCTTCACAAAAACCAAATACCA
**HOXD10**	Forward<br>Reverse	GGTCGGTTGTTTGTAGCGC<br>CTCGCAAATCACGTACTCCG	GTTGGGTTGGTTGTTTGTAGTGT<br>CCTCCTCACAAATCACATACTCCA
**ITIH5**	Forward<br>Reverse	AGAATTTCGGGGATGCGGATC<br>CAACTATCCACGACGTCCTCG	TGTAGAATTTTGGGGATGTGGATT<br>AAACAACTATCCACAACATCCTCA
**RASAL3**	Forward<br>Reverse	CGTTGGAGTTCGCGTTGTTC<br>CACCCTACTCCCCGAAACG	GGGTGTTGGAGTTTGTGTTGTTT<br>ACCAACCTCTAATCACTCAAATCCA
**CYP1B1**	Forward<br>Reverse	GGTTAGAGGTCGGTAGGTTGC<br>AAACGCTACTCTACGCTCCG	GTGGTTAGAGGTTGGTAGGTTGT<br>AAATTCCCACACACCTATCAAAACA

Gene expression analysis of CLBL1 cells treated with hypomethylating agents

A functional validation of microarray data was performed evaluating the mRNA expression restoration of 3 hypermethylated genes after the treatment of a canine B-cell lymphoma cell line (CLBL1: Rütgen et al., 2010), with hypomethylating agents. To this purpose azacytidine (AZA) and decitabine (DEC) were used. Among the hypermethylated genes, CADM1, CDH11 and ABCB1 were selected.

The CLBL1 cell line was maintained in T25 or T75 flasks as previously reported (Rütgen et al., 2010). Cells were seeded at a concentration of 3×10^5 cells/well in a 6-well flat bottom plate (Sarstedt Italia, Verona, Italy) and incubated for 72 h with AZA and DEC (Sigma-Aldrich, Milan, Italy) at the final concentration corresponding to their IC_{50} values (3.42 and 0.13 µM, respectively), determined by Alamar Blue test (Promega, Madison, USA). Due to its chemical instability, AZA dilution was freshly prepared every 24 h and added onto each well. Four independent experiments were performed.

At the end of the treatment, cells were collected and washed with PBS. Then, total RNA was extracted using the RNeasy® Mini Kit (Qiagen®, Hilden, Germany) and quantified with NanoDrop 1000 Spectrophotometer (Thermo Scientific, Waltham, Massachusetts, USA). One µg of total RNA was reverse-transcribed using the High Capacity cDNA Reverse Transcription kit (Life Technologies, Carlsbad, California, United States), according to the manufacturer's instructions.

For each target transcript, gene-specific primers encompassing one intron were designed (see Table S3). Two internal control genes (ICGs: GOLGA1 and CCZ1) previously published in Giantin et al. (2013) and Giantin et al. (2016) were selected.

The qPCR reaction was performed in a final volume of 10 µL, using 12.5 ng of cDNA, the Power SYBR Green PCR Master Mix (Life Technologies, Carlsbad, California, United States) and a

Stratagene Mx3000P thermal cycler (Agilent Technologies, Santa Clara, California, United States). Standard qPCR conditions were used, except for the analysis of CADM1 and CDH11, for which Ct values were acquired at 78°C to eliminate primer dimers contribution to the amplification plot. Different concentrations of forward (F) and reverse (R) primers were tested. The presence of specific amplification products was confirmed by dissociation curve analysis. For each qPCR assay, negative controls (with total RNA or water as template) and positive controls (the cDNA of 6 canine control lymph nodes) were run. Standard curves were obtained using the best performing primer concentration and serial dilutions of control lymph node cDNA. Each dilution was amplified in duplicate. The ΔΔCt method (Livak and Schmittgen, 2001) was used for the analysis of gene expression results.

Statistical analysis was performed using GraphPad Prism version 5.00 for Windows (GraphPad Software, San Diego, USA). Data were analysed using unpaired t-test. A *P* value < of 0.05 or less was considered as statistically significant.

Table S3. Primer pairs used for gene expression analysis (qPCR) in CLBL1

**Gene**		**Primer sequence 5’-3’**	**Primer concentration (nM)**
**CADM1**	Forward	GGTGAGGAGATTGAAGTGAACTG	50
	Reverse	TCCTCCACCTCCGATTTGC	300
**CDH11**	Forward	CATTAACGACAACCCTCCTGAG	300
	Reverse	CTGGATGACCGACGTTCCC	50
**ABCB1**	Forward	GACGTTGGGGAGCTTAACAC	300
	Reverse	CGCCAATTCCTTCATTGATT	600

SUPPLEMENTAL RESULTS

Data preprocessing

The combining Loess and Quantile normalization pre-process used to normalize the methylation data, was able to overcome some pitfalls of the data distribution. In Fig. S3, the position of Dog#19 is shown both in the PCA and MA plots with respect to the median across the samples, highlighting the difference obtained by the two normalization approaches. In Fig S3 A. and C., only Quantile normalization is applied, whereas in Fig S4 B. and D. Loess approach is combined with Quantile. In the latter, Dog#19 clustered with the cDLBCLs group. Indeed, this sample showed the most evident dye-bias (Figure S4) generated by the fact that Cy5 signal was altered by the experimental enrichment step generating an additional bias compared to the Cy3-labeled reference. This trend was observed in more than half of the samples of the dataset. The Loess normalization step was able to determine the adjustment of this bias using the information from each dye. Furthermore, since Quantile normalization assumes a common distribution of data, the Loess-normalized data were characterized by a more similar between-array distribution compared to the raw data (Figure S5), thus allowing Quantile-normalization to have the best fit.

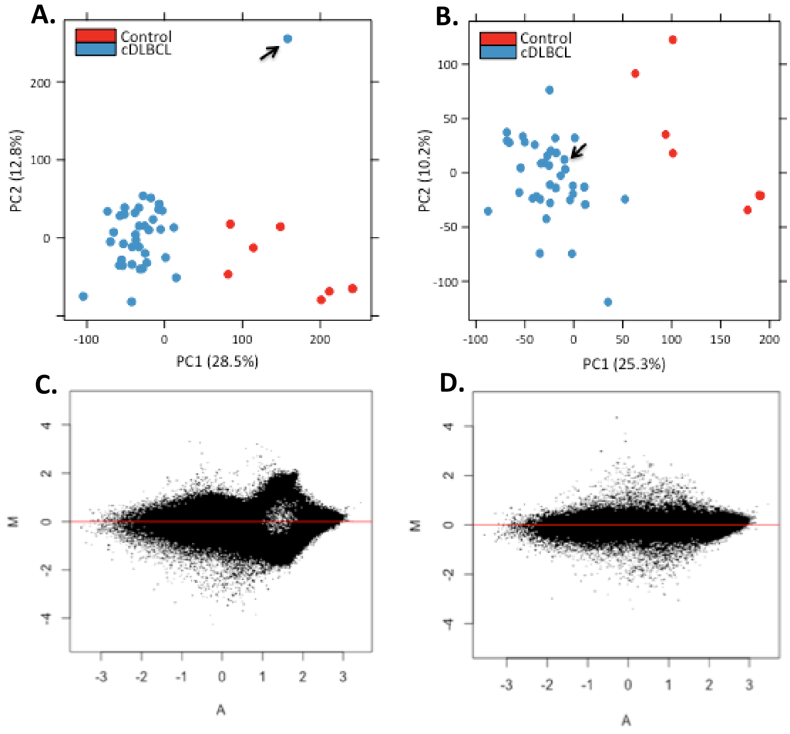

Figure S3. Differences between Quantile-only and Loess-plus-Quantile normalization on methylation data. PCA analysis on cDLBCL and control samples (upper panel) and MA plots on sample Dog#19 (lower panel), showing differences between Quantile normalization applied directly on raw data (left panel) and the same Quantile normalization applied on Loess-normalized data adjusted for the dye-bias. Arrows in the PCA plots indicate the positions of Dog#19.

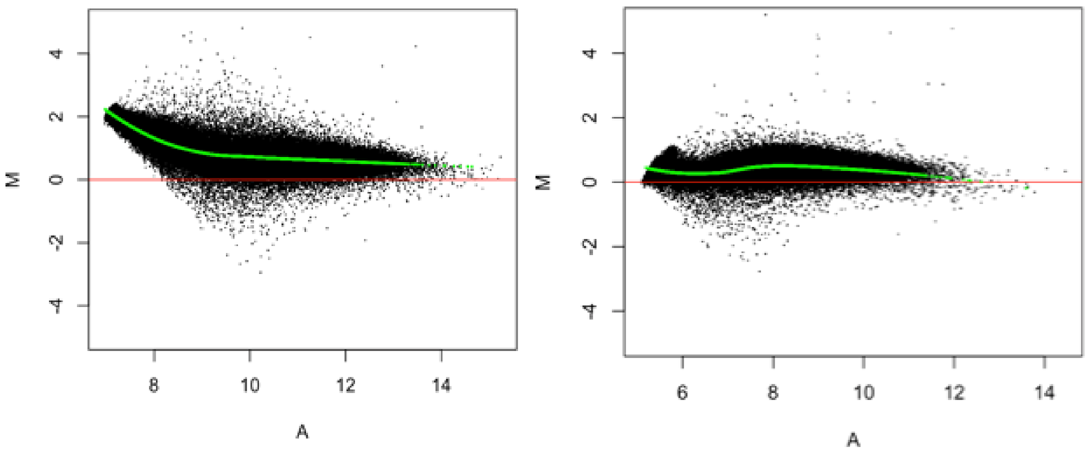

Figure S4. Between-array MA plot of cDLBCL Dog#19, separating Cy5-dye (right panel) and Cy3-dye (left panel) signals.

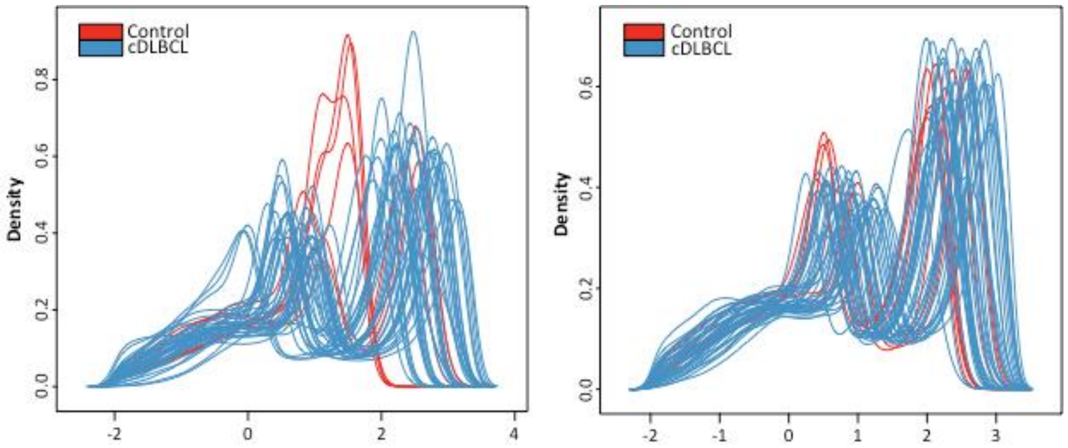

Figure S5. Density plots of the arrays on raw (left panel) and Dye-bias normalized (right panel) data.

Microarray data technical validation

In order to quantify the ratio of methylated to unmethylated alleles, the ΔCT (=CT_Meth – CT_NoMeth) value was determined (Table S3) as described by Zeschnigk et al. (2004). The Mann-Whitney test comparing Meth/No-Meth primer pairs showed a significant hypermethylation between the two groups for HOXD10, RASAL3 ($p<0.001$), CYP1B1 and ITIH5 ($p<0.01$), while FGFR2 was only marginally significant ($p=0.07$).

Table S3. ΔCT values calculated, for each target gene, on cDLBCL and Control samples

cDLBCL	FGFR2	HOXD10	ITIH5	RASAL3	CYP1B1
15	3.56	-1.94	-6.51	-3.99	3.95
30	2.94	-1.07	-5.45	-3.51	5.73
20	-1.48	-1.45	-5.07	-3.14	-1.02
33	5.04	-0.69	-2.77	-3.08	5.21
14	1.16	-2.61	-4	-4.17	3.5
24	5.45	-1.08	-3.27	-2.96	0.08
12	5.62	-2.65	-	-4.5	4.73
19	-0.16	0.98	0.2	-2.28	1.43
6	1.94	-0.35	-3.36	-4.07	2.84
38	7.25	0	-2.56	-6.36	1.23
32	7.12	-2.03	-2.92	-4.44	0.55
18	5.82	0.27	-3.79	-3.64	3.95
34	9.31	2.37	-3.8	-4.03	3.29
**Controls**					
Ctrl#7	4.4	3.47	-0.89	-0.34	4.9
Ctrl#4	7.13	4.4	-1.69	-1.07	5.87
Ctrl#8	8.36	4.03	-2.65	-2.64	5.73
Ctrl#2	8.6	5.57	-2.22	-2.57	6.51
Ctrl#1	6.39	3.72	-1.96	-1.49	5.56

Microarray data functional validation

All primer pairs for gene expression analysis had an acceptable efficiency (range 90 % ÷ 110 %), and a slope in the range of -3.6/-3.1. The main features of the validated qPCR assays are reported in Table S4.

Table S4. Main features (slope, efficiency, R^2, dynamic range) for each qPCR assay.

Gene	Slope	Efficiency (%)	R^2	Dynamic range (Ct)
**CADM1**	-3.36	98.4	0.99	24.48 – 34.60
**CDH11**	-3.47	94.1	0.99	22.75 – 36.62
**ABCB1**	-3.18	106.4	0.99	28.79 – 36.17

The effect of AZA and DEC treatment on CADM1, CDH11 and ABCB1 mRNA expression are summarized in Figure S6-S8, respectively.

Selected genes were all constitutively and highly expressed in the control lymph nodes, while in the B-cell lymphoma cell line (CLBL1) they were almost completely silenced. Following the treatment with AZA, the mRNA expression was significantly restored (P<0.05 or less). Conversely, DEC affected the re-expression of ABCB1 (P<0.01), while did not exert any effect on CADM1 and CDH11 (data not shown).

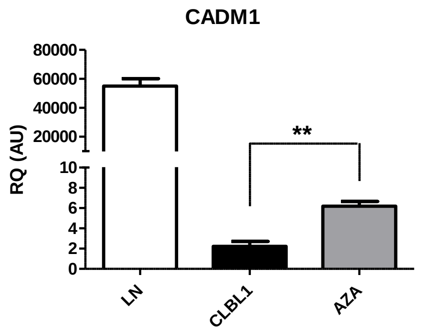

Figure S6: CADM1 mRNA expression in control lymph nodes (LN), CLBL1 cells alone and treated with azacytidine (AZA, 3.42 µM). RQ values are expressed in arbitrary units (AU) as means ± SEM.

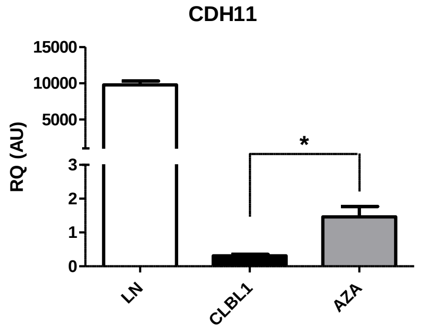

Figure S7: CDH11 mRNA expression in control lymph nodes (LN), CLBL1 cells alone and treated with azacytidine (AZA, 3.42 µM). RQ values are expressed in arbitrary units (AU) as means ± SEM.

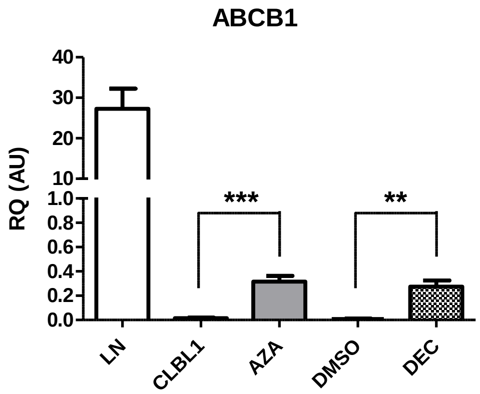

Figure S8: ABCB1 mRNA expression in control lymph nodes, CLBL1 cells alone and treated with the vehicle (DMSO 0.1%), azacytidine (AZA, 3.42 µM) and decitabine (DEC, 0.13 µM). RQ values are expressed in arbitrary units (AU) as means ± SEM.

SUPPLEMENTAL REFERENCES

Alexa A, Rahnenfuhrer J, and Lengauer T. Improved scoring of functional groups from gene expression data by decorrelating go graph structure. *Bioinformatics* **2006**; 22:1600–1607.

Hansen KD, Timp W, Bravo HC, Sabunciyan S, Langmead B, McDonald OG, *et al.* Increased methylation variation in epigenetic domains across cancer types. *Nat Genet* **2011**; 43: 768-775.

Giantin M, Vascellari M, Lopparelli RM, Ariani P, Vercelli A, Morello EM *et al.* Expression of the aryl hydrocarbon receptor pathway and cyclooxygenase-2 in dog tumors. *Res Vet Sci.* **2013**; 94:90-9.

Giantin M, Baratto C, Marconato L, Vascellari M, Mutinelli F, Dacasto M *et al.* Transcriptomic analysis identified up-regulation of a solute carrier transporter and UDP glucuronosyltransferases in dogs with aggressive cutaneous mast cell tumours. *Vet J*. **2016**; 212:36-43.

Hernández H.G., Tse M.Y., Pang S. C., Arboleda H., Forero D.A. Optimizing methodologies for PCR-based DNA methylation analysis. *BioTechniques* **2013**; 55:181-197

Livak KJ and Schmittgen TD. Analysis of Relative Gene Expression Data Using Real-Time Quantitative PCR and the $2^{-\Delta\Delta CT}$ Method *Methods* **2001**; 25:402–408.

Rütgen BC, Hammer SE, Gerner W, Christian M, de Arespacochaga AG, Willmann M *et al.* Establishment and characterization of a novel canine B-cell line derived from a spontaneously occurring diffuse large cell lymphoma *Leuk Res* **2010**; 34:932–938.

Shaffer AL, Wright G, Yang L, Powell J, Ngo V, Lamy L *et al.* A library of gene expression signatures to illuminate normal and pathological lymphoid biology. *Immunol Rev* **2006**; 210:67-85.

Subramanian A, Tamayo P, Mootha VK, Mukherjee S, Ebert BL, Gillette MA, *et al.* Gene set enrichment analysis: a knowledge-based approach for interpreting genome-wide expression profiles. *PNAS* **2005**; 102:15545–15550.

Szklarczyk D, Franceschini A, Wyder S, Forslund K, Heller D, Huerta-Cepas J, *et al.* STRING v10: protein-protein interaction networks, integrated over the tree of life. *Nucleic Acids Res* **2015**; 43:D447-52

Wilkerson MD and Haynes DN. ConsensusClusterPlus: a class discovery tool with confidence assessments and item tracking. *Bioinformatics* **2010**; 26(12):1572-1573.

Zeschnigk M, Böhringer S, Price EA, Onadim Z, Masshöfer L, Lohmann DR. A novel real-time PCR assay for quantitative analysis of methylated alleles (QAMA): analysis of the retinoblastoma locus. *Nucleic Acids Res* **2004**; 32(16):e125.

SUPPLEMENTAL FIGURES

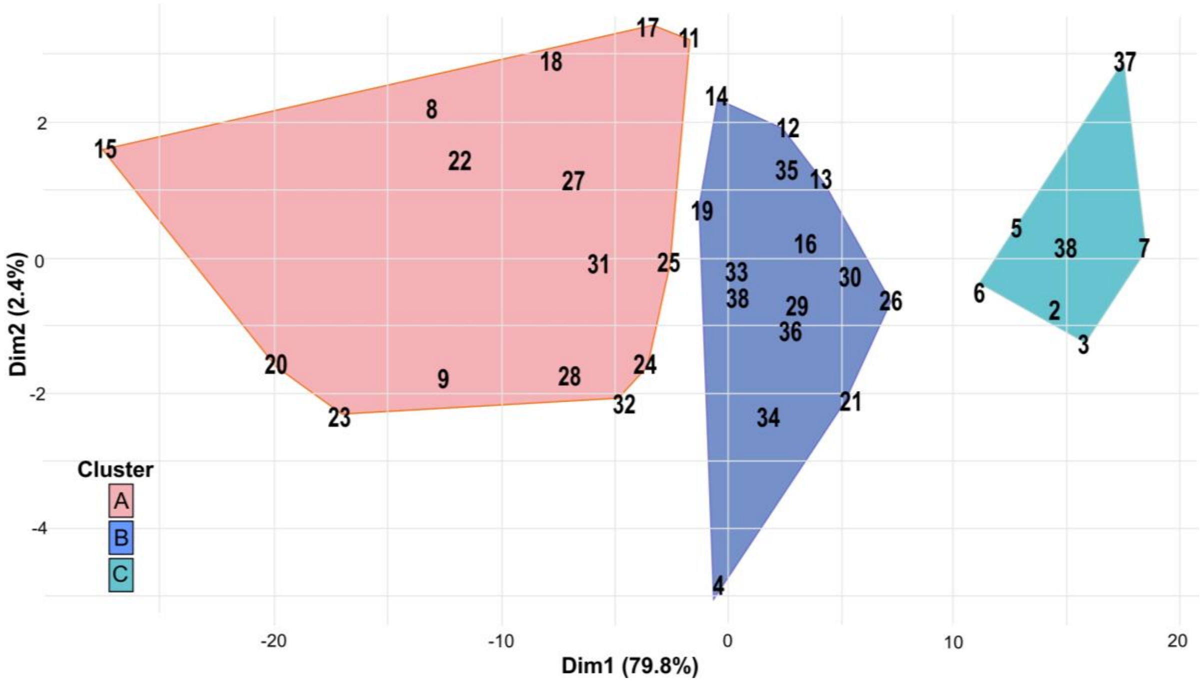

Figure S9. PCA plot on cDLBCL samples based on MVPs. Colors correspond to the clusters identified by the sequences highly correlated with the first principal component.

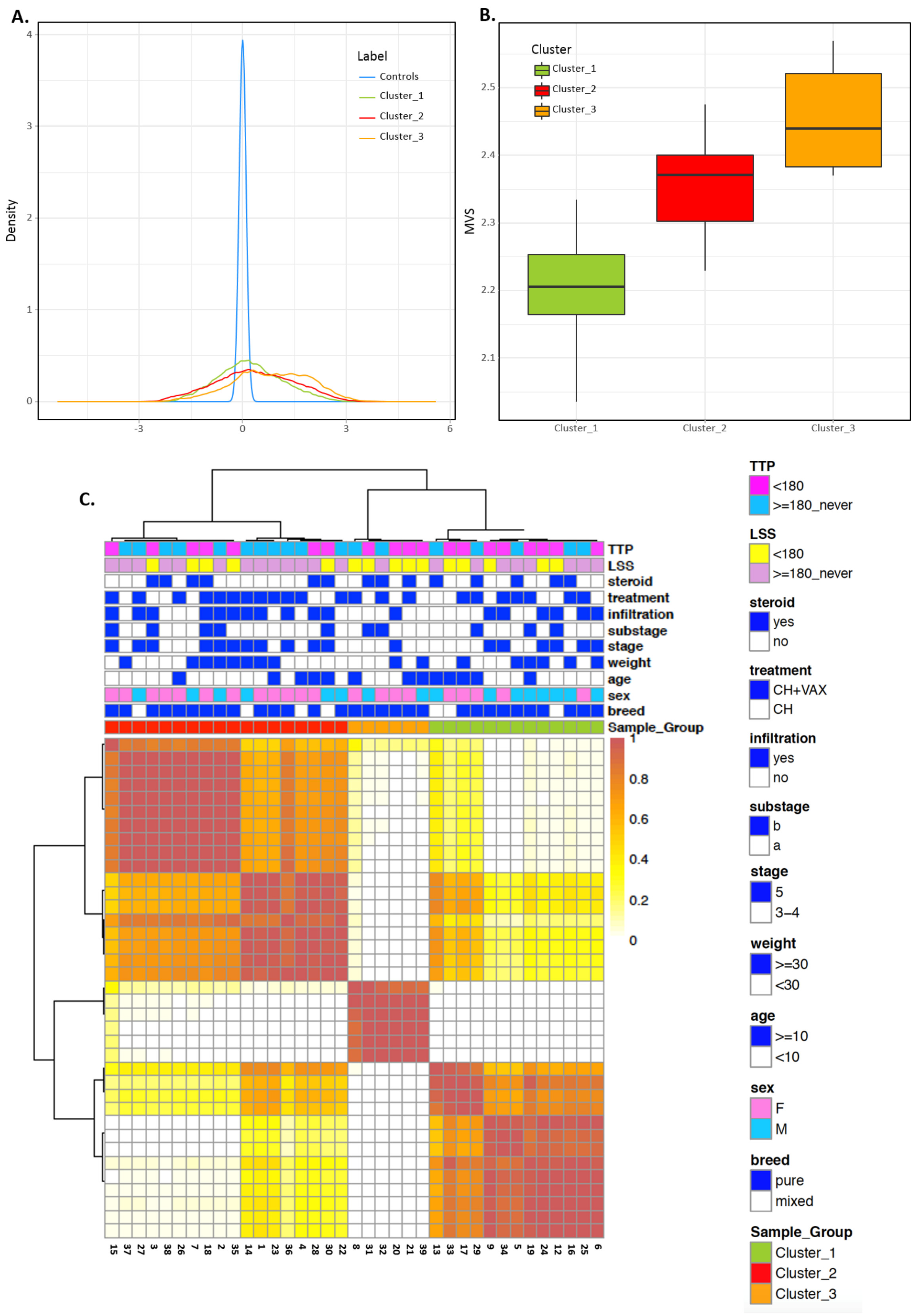

Figure S10: Consensus hierarchical clustering on the first 2,000 sequences showing the highest median absolute deviation of the MVPs across cDLBCLs A. Density function of MVPs of cDLBCL clusters compared to those of controls, **B.** Boxplot of the MVS by cluster. **C.** Heatmap for consensus matrix (K=3)

SUPPLEMENTAL TABLES

Excel file Supplementary Table S2

Table S2.xls Differentially hyper- and hypo-methylated sequences on cDLBCL samples with respect to normal lymph nodes. Columns H-I report the median methylation level across cDLBCL and normal samples, respectively. Columns K-R report the overlapping Refseq (K-N) and Ensembl (O-R) transcripts, considering for each transcript the following genomic locations: exon, intron, 5'-UTR, 3'-UTR, "proxUP" (i.e. until 2kb upstream from transcription start site), "upstr" (i.e. from 2kb to 10kb upstream from transcription start site), "proxDOWN" (i.e. until 2kb downstream from transcription start site). Columns S-V: nearest Refseq or Ensembl transcripts calculating the distance from the transcription start site (TSS).

Excel file Supplementary Table S3

Table S3.xls List of significant probes/genes obtained after categorical division of the Beta values in two classes. Column A reports the exact genomic location of the probe and Column B reports the gene symbol.

Excel file Supplementary Table S4

Table S4.xls Significantly enriched GO terms and KEGG pathways for the differentially hyper- and hypo-methylated sequences on cDLBCL samples with respect to normal lymph nodes.

Excel file Supplementary Table S5

Table S5.xls Significantly enriched gene signatures highlighted by Gene Set Enrichment Analysis (GSEA) on the entire dataset of probes.

Excel file Supplementary Table S6

Table S6.xls Sequences showing differential methylation variability on one or more clinical factors across the cDLBCL samples. Columns G-N report the overlapping Refseq (G-J) and Ensembl (K-N) transcripts, considering for each transcript the following genomic locations: exon, intron, 5'-UTR, 3'-UTR, "proxUP" (i.e. until 2kb upstream from transcription start site), "upstr" (i.e. from 2kb to 10kb upstream from transcription start site), "proxDOWN" (i.e. until 2kb downstream from transcription start site). Columns O-R: nearest Refseq or Ensembl transcripts calculating the distance from the transcription start site (TSS).

Excel file Supplementary Table S7

Table S7.xls Results from multivariate linear regression analysis, investigating different combination of clinical factors across the cDLBCL samples. Columns H-R: clinical factors considered for the analysis; “1” indicates the presence of that factor in the linear regression model. Columns S-Z report the overlapping Refseq (S-V) and Ensembl (W-Z) transcripts, considering for each transcript the following genomic locations: exon, intron, 5’-UTR, 3’-UTR, “proxUP” (i.e. until 2kb upstream from transcription start site), “upstr” (i.e. from 2kb to 10kb upstream from transcription start site), “proxDOWN” (i.e. until 2kb downstream from transcription start site). Columns AA-AD: nearest Refseq or Ensembl transcripts calculating the distance from the transcription start site (TSS).
